# Treatment of Chronic Delta Hepatitis

**DOI:** 10.5812/kowsar.1735143X.759

**Published:** 2011-09-01

**Authors:** Mario Rizzetto

**Affiliations:** 1 Department of Gastroenterology, Molinette, University of Torino, Torino, Italy

**Keywords:** Treatment, Chronic hepatitis D

Hepatitis D virus (HDV) infections remain a worldwide health problem. However, the clinical impact of these infections is often underestimated, and therapeutic options for the infections are often unexploited. Gulsun et al. have addressed both these issues in the paper "Treatment of chronic hepatitis D; a 9 years retrospective analysis" published in this issue of Hepatitis Monthly.

The present study was conducted in Diyarbakir, Eastern Turkey, where chronic hepatitis D is one of the most prevalent liver diseases. HDV causes liver failure due to cirrhosis and hepatocellular carcinoma in a significant proportion of hepatitis B surface antigen (HBsAg) carriers. Over 8 years, The authors enrolled 46 patients with florid hepatitis D. These patients showed the typical features of HDV infections; most of them were young adult men with concomitant hepatitis B virus (HBV) infections that were positive for the antibody to hepatitis B antigen and not for the HBe antigen.

Treatment of hepatitis D is a formidable problem because HDV totally depends on the replication machinery of the hepatocytes for its replication and has no specific transcriptases that can be targeted by antivirals. Efforts to target the concomitant HBV infection were in vain, and neither lamivudine nor adefovir could ameliorate hepatitis D or abrogate the underlying HBV infection. The authors used the only currently available therapeutic agent, pegylated interferon-alpha (PEG-IFN-α), for treating patients. The use of INFs for treating hepatitis D was introduced long back in 1980 after considering the efficacy of cytokines in treating hepatitis B. INF use is still based on the experiences from common practice rather than conclusive evidence from clinical trials, and many questions regarding the use of INFs in the management of HDV patients remain unanswered; some of these were answered in the study by Gulsun.

These authors determined whether long-term therapy is more efficacious than 1-year therapy. Their results showed that both long-term and 1-year therapy had the same outcomes; this finding is similar to that of another Turkish study [1]. Therefore, if there is no virologic response, then therapy should be discontinued after a year; this will help avoid causing unnecessary inconvenience to patients and reduce the costs incurred by health agencies.

Importantly, in this study, PEG-INF-α treatment was more successful than in most previous studies; it achieved a sustained virologic response (SVR) rate of 41%. The good therapeutic outcome obtained with PEG-IFN-α-2a in this study may be attributable to the relatively young age of the patients (median age, 35 years) because young age is correlated with less fibrosis, and in previous studies, fibrosis has been shown to reduce the efficacy of IFN-α treatment. The age-specific analysis has indeed shown that the decline of viremia under therapy was more significant and the decrease of the Knodell score was higher in patients younger than 35 than in those older than 35, and in keeping with a better liver function in less advanced disease, side effects were minor, and only 5 patients required episodic reductions of therapy. Among the other studies that reported outcomes of PEGIFN treatment, a French study [2] included the highest proportion of non-cirrhotic patients with less advanced diseases and reported the highest SVR rate (43%) for patients treated with PEG-IFN-α-2b. Gulsun et al. reported a higher rate of relapse in patients who originally had elevated serum HDV-RNA and HBsAg levels. Further analysis of such patients in terms of their age, initial symptoms and their response to 1-year and 2-year long therapy would be interesting.

Though the data obtained by Gulsun et al. are encouraging, the clinical significance of the SVR rates after IFN treatment achieved in this and other studies is unclear. Hepatitis D is sustained by a double viral infection, which makes the evaluation of therapeutic endpoints more complex. The long-term significance of achieving virologic and clinical therapeutic targets other than absence of HBsAg has not been assessed so far. The viral titers during infections caused by HDV are much lower than those during infections caused by HBV and hepatitis C virus. The infectivity of HDV is very high and titers of the virus well below the detectability threshold of current assays might recapitulate cell to cell transmission of HDV within the liver and reactivate infection if the HBsAg is around. Thus, a long-term follow-up study of our patients is needed to clearly establish the therapeutic role of PEG-IFN in treating chronic hepatitis D.
